# Costs and cost-effectiveness of type 2 diabetes management in Sub-Saharan Africa: a systematic review

**DOI:** 10.1186/s13561-026-00777-1

**Published:** 2026-04-22

**Authors:** Assegid Hellebo, Andre P. Kengne, Olufunke A. Alaba

**Affiliations:** 1 Health Economics Unit, School of Public Health, Faculty of Health Sciences, University of Cape Town, Anzio Rd, Observatory, Cape Town, 7925 South Africa; 2 Department of Medicine, University of Cape Town and Non-Communicable Diseases Research Unit, South African Medical Research Council (SAMRC), Francie Van Zijl Dr, Parow Valley, Cape Town, 7501 South Africa

**Keywords:** Sub-Saharan Africa, Healthcare, Costs, Cost-effectiveness, QALYs, DALYs, T2D, Type 2 diabetes management

## Abstract

**Background:**

Type 2 Diabetes (T2D) is rapidly increasing in Sub-Saharan Africa (SSA), with related health and financial implications. Knowledge of the costs and cost-effectiveness of managing diabetes is needed to increase awareness, improve resource allocation and enhance the use of evidence-based decision-making. Accordingly, this study sought to synthesise and critically review evidence on the costs and cost-effectiveness of managing T2D in Sub-Saharan Africa (SSA).

**Methods:**

A systematic search was conducted in multiple databases, including PubMed-Medline, Africa-Wide Information, Web of Science, Cochrane Library, CINAHL, Scopus, and Google Scholar, to identify studies published between January 2010 and December 2023; the searches were updated in June 2024. All cost data were converted to 2024 United States dollars (US$) using appropriate inflation adjustments and exchange rates. The review protocol was registered with PROSPERO (CRD42022300580).

**Results:**

The search yielded 4137 unique abstracts, and 18 studies were included for review, most originating from Nigeria. Most studies assessed costs from a provider perspective, focusing on direct medical costs (e.g., outpatient consultations, medications, diagnostic and monitoring tests, and follow-up care). Reported costs of T2D management were substantial across settings. Annual costs ranged from US$337.50 for basic medical care of uncomplicated T2D to US$2330.74 in total provider cost per patient. The highest burden was reported among individuals in the low-income quantile. Only five studies assessed cost-effectiveness, most using modelling and simulation approaches. T2D management programmes can be cost-effective, but the evidence remains limited.

**Conclusions:**

This review identified substantial variation in methods, perspectives, and cost components across T2D cost studies in SSA, which limits direct comparability. Improving methodological transparency and reporting, alongside addressing data-collection gaps, would strengthen the evidence base. Further context-specific economic evaluations, particularly those incorporating affordability, implementation feasibility, and budget impact, are needed to support priority setting and resource allocation for T2D management in SSA.

## Introduction

Diabetes mellitus is a leading public health challenge worldwide [1, 2]. Once regarded as a disease of the affluent, it has become a significant and growing health challenge in low-and middle-income countries (LMICs) [3, 4]. Although diabetes was historically less prominent in public health discussions in Sub-Saharan Africa (SSA), its prevalence has increased substantially over the past three decades. However, in 2021, it was one of the leading causes of non-communicable disease (NCD) deaths, contributing to 416,000 deaths in SSA [3]. A high number of diabetes-related deaths occurs among economically productive adults below the age of 60 years [4, 5]. The International Diabetes Federation (IDF) estimates that the number of individuals living with diabetes in the SSA region will be 55 million in 2045 from 24 million in 2021 [5].

Around 60% of individuals with diabetes in SSA remain undiagnosed, making it a notable characteristic of the disease in the region [6], highlighting the need for improved screening and diagnostic efforts to ensure timely detection and proper management. Among the countries located in SSA, more than 50% of the working-age adults with diabetes live in South Africa, Nigeria, the Democratic Republic of Congo and the Federal Republic of Ethiopia, the region’s most prominent countries [7]. The increase in diabetes figures and linked morbidities and mortality impose an immense financial burden on households, healthcare systems and the states [7, 8]. According to the IDF, the SSA region’s healthcare expenditure due to diabetes was US$3.4 billion in 2015 and is estimated to reach US$5.5 billion in 2040 [3, 5].

Diabetes is potentially preventable, and once present, appropriate management can halt or delay related complications. Diabetes is a chronic metabolic condition that is often difficult to manage and requires long-term clinical care [9, 10]. To curb the reported low self-management, individual patient-level, clinician, or organisation-targeted interventions are recommended to improve care delivery, check-up rates and glycaemic management among patients with T2D [9, 10]. The World Health Organisation (WHO) guidelines recommend managing the disease at its early stage [11]. Such management often involves an improved diet, taking medications and monitoring blood glucose. The intended primary outcome is to manage blood glucose levels and minimise the effect of risk factors including body mass index (BMI) and blood pressure (BP), to avoid further complications and early death [10, 11].

Managing T2D often carries a high burden of direct (medical and non-medical) and indirect costs. Costs are resources incurred by providing or attaining healthcare goods and services to maintain or implement a person or population health programme [12]. The direct medical costs include hospital stays, laboratory tests, and doctor visits. At the same time, non-medical costs also include travel for treatment and care for loved ones taking care of the patient [13]. The indirect costs include lost productivity for the people involved in the patient’s care and work absenteeism [14].

Cost-effectiveness is one branch of economic evaluation that examines the costs and health outcomes of one or more interventions [14, 15]. When assessing two interventions or programmes, intervention/programme ‘A’ is deemed cost-effective if its cost per unit of effectiveness is lower than that of intervention/programme ‘B’. The cost-effectiveness of interventions may also be measured using a threshold or benchmark of a region/country per the gross domestic product (GDP) per capita [14, 15]. Past studies have used the cost-effectiveness thresholds established by the Commission on Macroeconomics and Health [16, 17]. This is the cost per averted disability-adjusted life years (DALY) of less than three and less than one times the country’s GDP per capita for cost-effective and highly cost-effective interventions, respectively [14, 15]. Such health outcomes are measured through differences in quality of life (QoL), DALYs, quality-adjusted life years (QALYs) and return on investment (ROI) [17].

T2D demands continuous clinical care and management, which consumes significant healthcare resources [17, 18]. For a region where resources are constrained and health financing heavily depends on out-of-pocket payments, the authorities battle with the costs of managing diabetes [8]. Therefore, conducting economic evaluation research in SSA is crucial to raise awareness and potentially improve resource allocations. Thus, this study aimed to synthesise available evidence on the costs and cost-effectiveness of managing T2D and its complications in SSA. We conducted this systematic review to (i) provide costs of T2D management in SSA and (ii) assess whether lifestyle interventions for T2D management are cost-effective in SSA or not.

## Materials and methods

### Searches and search strategies

The literature search followed the Preferred Reporting Items for Systematic Reviews and Meta-Analyses (PRISMA) guidelines [19]. We systematically searched the following databases: Medline (via PubMed), Africa Wide Information, Web of Science, Cochrane Library, CINAHL, Scopus, and Google Scholar. Search strategies and key terms were developed based on the study’s target population, interventions, comparators, and outcomes (PICOS) and validated by an experienced university librarian. A summary of the search strategies and terms is provided in the Appendix.

Searches were conducted in January 2023 and updated in June 2024, restricted to English and French language publications. We searched for studies published between January 2010 and December 2023. All search results were imported and merged across databases, and duplicates were removed using Rayyan software [20]. Titles and abstracts were screened for relevance, followed by full-text assessment to determine eligibility according to predefined inclusion criteria. The first author conducted the initial screening and data extraction, with verification by an independent reviewer. Disagreements were resolved through consultation with a third reviewer.

### Inclusion criteria

When undertaking the literature review, PICOS was used to guide eligibility criteria, where we used the relevant elements to decide on the inclusion and exclusion measures [19].

We included studies that (i) from provider or patient perspective involved at least one African country, (ii) presented original research findings on the costs or cost-effectiveness of type 2 diabetes (T2D) interventions, and (iii) were published in peer-reviewed journals. Eligible studies focused on T2D patients and/or their caregivers, given that T2D is the most prevalent form of diabetes in sub-Saharan Africa.

The primary outcomes of interest were the costs and cost-effectiveness of T2D interventions, assessed through measures such as incremental cost per unit of health benefit. Reported outcomes included cost per quality-adjusted life year (QALY) gained, cost per disability-adjusted life year (DALY) averted, and improvements in health-related quality of life (HRQoL).

### Exclusion criteria

We excluded studies that (i) lacked sufficient data on costs or cost-effectiveness, (ii) were non-original research such as reviews, commentaries, or qualitative studies, (iii) were duplicate or overlapping publications, and (iv) were unpublished grey literature or academic theses. Grey literature and theses were excluded to prioritise peer-reviewed evidence with transparent methodological reporting and to improve comparability across included studies.

### Data extraction

The latest Cochrane Handbook guided data extraction for systematic reviews of interventions [21]. Using an Excel spreadsheet, essential information from each study, including population size, characteristics of the participants, data collection methods, study perspective, study country, duration of the intervention, active and maintenance phases, details of intervention procedures, glycated haemoglobin (HbA1c), blood pressure (BP) outcomes and outcome measurements (QALYs, DALYs) were extracted.

A standardized data extraction template was developed and piloted on a subset of included studies prior to full data extraction to ensure clarity and consistency in capturing study variables. Data extraction was conducted by the first author and independently verified by a second reviewer. Any discrepancies were discussed and resolved through consensus.

All reported costs were standardized to 2024 United States dollars (US$), as 2024 represented the most recent year of data synthesis. For studies reporting costs in local currencies, we first adjusted the original values for inflation to the costing year using country-specific consumer price indices (CPIs). We then converted the inflation-adjusted costs to US$ using official exchange rates from the World Bank and International Monetary Fund for the corresponding year. Where exchange rates were not reported, we used the average annual rates for the costing period obtained from the Forex (FX) currency converter [22].

For studies conducted across multiple years (e.g., November 2009-January 2010), the final year of data collection was treated as the costing year. This approach ensured consistency and comparability of all cost estimates across studies.

Given the substantial heterogeneity across studies in terms of intervention types, costing perspectives, study designs, and outcome measures, quantitative pooling was not feasible. Instead, a narrative synthesis approach was used. Studies were grouped and summarised according to intervention type, costing perspective (provider, patient, or societal), and reported outcomes (cost of illness or cost-effectiveness measures such as cost per QALY or DALY). This approach allowed comparison of economic findings across studies while identifying patterns and differences across settings.

## Results

### Results of the search

**Fig. 1:**
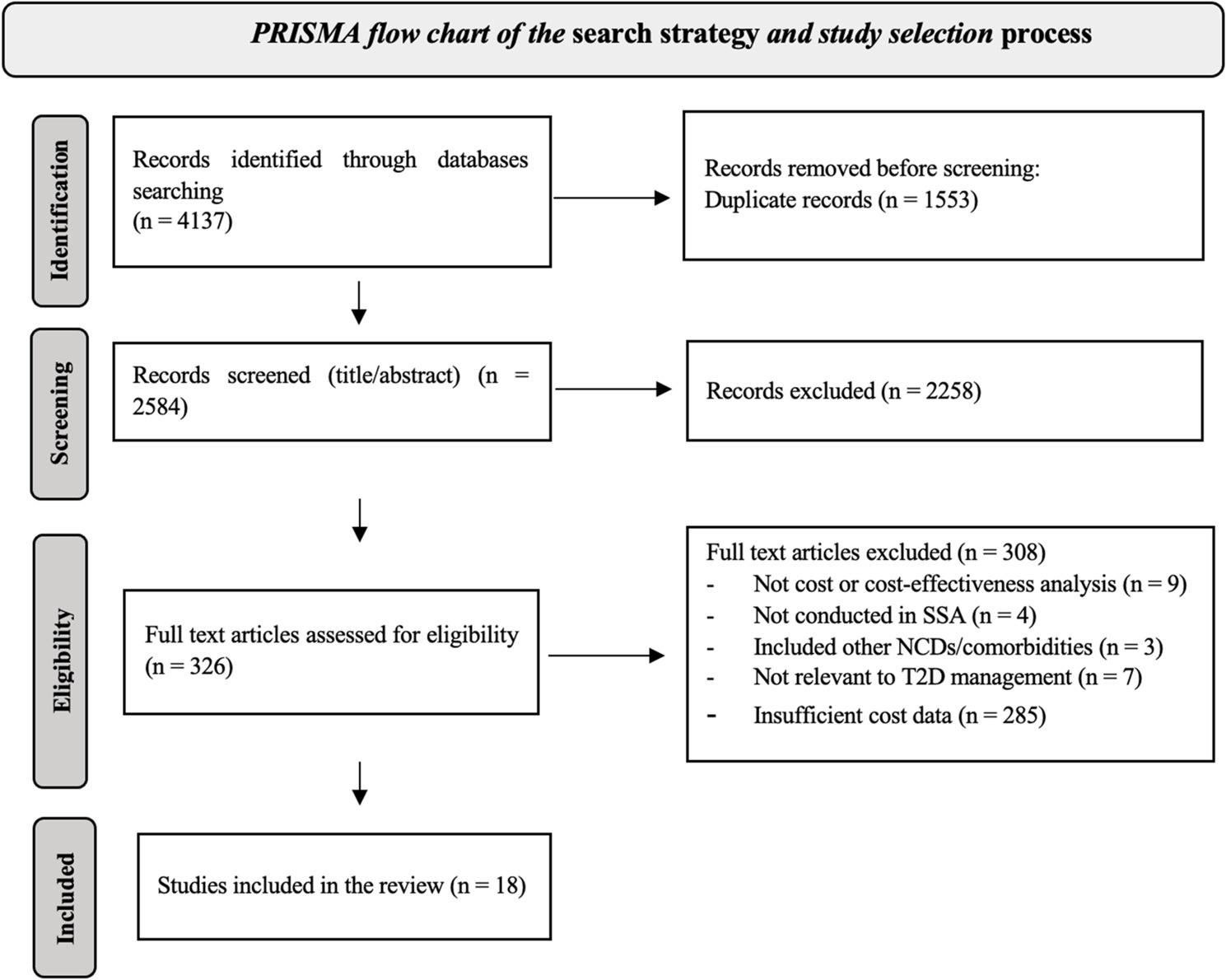
PRISMA flow chart of the search strategy and study selection process

A total of 4137 unique abstracts were screened, and 18 studies were included: 13 studies investigated the cost of T2D management, and five performed cost-effectiveness analysis (CEA), as illustrated in Fig. 1. A summary of the study characteristics, cost estimates, and cost-effectiveness outcomes is presented in Tables 1 and 2.

**Table 1:** Summary of the methods used and cost-of-illness / cost analysis studies (*n* = 13)

Author/s and Year	Country	Study design and/or study method	Target population and/or sample size	Duration/ analytical time horizon	Comparator	Intervention type and/or cost items	Intervention effectiveness outcome/s	Perspective	Cost in 2024 US$ and unit of cost
Abduganiyu & Fola (*2014*) [17]	Nigeria	Retrospective case-note review and cost analysis	T2D patients attending the University of Maiduguri Teaching Hospital diabetes clinic (*n* = 96); cost analysis using prescription dataset (*n* = 1200)	One year	Branded vs. generic antidiabetic medicines	Direct cost of T2D management and cost comparison of generic vs. branded antidiabetic therapy	Not reported	Societal	US$799.24 per patient per year (direct healthcare cost); US$0.32 per generic defined daily dose (DDD) vs. US$0.50 per branded DDD
Fadare et al. (2015) [23]	Nigeria	A cross-sectional study carried out using the eight-item Morisky Medication Adherence Scale (MMAS-8)	Type 2 diabetes patients who had been on medication for at least six months attending the medical outpatients’ diabetes clinic (*n* = 129)	Three months	None	Assessing direct cost and the level of adherence to antidiabetic drugs among outpatients in a teaching hospital	Not reported	Patients	US$55.35 per month/patient
Ipingbemi & Erhun (2015) [18]	Nigeria	Retrospective study using case files	Type 2 diabetes patients attending secondary healthcare facility (*n* = 52)	One year	None	Direct and indirect costs of diabetes	Not reported	Patients	US$508.37 per annum/patient
Ipingbemi et al. (2021) [24]	Nigeria	A quasi-experimental study	Type 2 diabetes patients attending the endocrinology outpatient clinics in two-tertiary facilities (*n* = 201)	One year	The control group continued to receive the usual care	The direct cost of disease management is for pharmacist-led educational intervention to resolve adherence discrepancies.	Significant reduction in the HbA1c and systolic blood pressure as well as improvement in weekly physical activity	Patients	US$333.0 ± US$118.4 per annum/patient
Okoronkwo et al. (2015) [25]	Nigeria	Cross-sectional descriptive survey	Type 2 diabetes patients aged between 31 and 65 years managed at a tertiary health institution (*n* = 308)	One year	None	Direct cost borne by patients	Not reported	Patients	US$502.63 per annum/patient
Alouki et al. (2017) [26]	Mali	Cross-sectional study: Participants were randomly selected from registries.	Type 2 diabetes patients attending three public hospitals, pharmacies, private clinics and private pharmacies (*n* = 500)	One year	Costs for basic medical care of uncomplicated diabetes	Costs for basic medical care of complicated chronic diabetes	Not reported	Patients	Median annual expenditure is US$337.5 per patient with complications while US$249.2 without complications.
Bermudez-Tamayo et al. (2017) [27]	Mali	Observational retrospective case-control	Randomly selected subjects from diabetes registries (*n* = 500)	Three months	Those with no diabetes (*n* = 500)	Direct and indirect costs (productivity losses by patients and caregivers and absenteeism)	Not reported	Societal	US$346.01 for three months/patient
Masis et al. (2023) [28]	Kenya	A retrospective cost analysis combining micro- and gross costings	Uncomplicated type 2 diabetes patients (*n* = 331)	One year	None	Direct and indirect medical costs of outpatient treatment	Not reported	Provider	US$46.86/ (visit consultation, RBS test, HbA1c test and the annual pharmacotherapy regimens)
Obakiro et al. (2021) [29]	Uganda	Retrospective, cross-sectional	Diabetes patients attending the outpatient medical clinic (*n* = 2612)	One year	None	Outpatient medical clinic costs	Not reported	Patients	US$12.59 Cost/patient/prescription
Quaye et al. (2015) [30]	Ghana	Descriptive cross-sectional, random selection	Patients with type 2 diabetes (*n* = 304)	One year	None	Case management carried out in clinics	Not reported	Provider	US$498.40 per annum/patient
Wargny et al. (2018) [31]	Senegal	A controlled open clinical trial with a randomised time allocation of the intervention	People with type 2 diabetes who have signed up, for free, to the mD iabetes programme residing in northwest and south of Dakar centres (*n* = 186)	Six months	None	Sending daily Short Message Service (SMS) for three months	HbAc1 decreased over the three months after having stopped in both centres	Provider	US$3.80 per 3 months/campaign patients
Erzae et al. (2019) [32]	South Africa	Bottom-up (person-based) using cost of illness (COI) approach	Type 2 diabetes patients who were diagnosed, treated and controlled in 2018 (*n* = 55,000)	One year	Estimated direct medical costs for basic chronic treatment	Actual expenditures of diabetic patients	Not reported	Provider	US$986.6 per annum/patient
Opperman & De Klerk (2021) [33]	South Africa	Retrospective Review of Membership Claims	Type 2 diabetes patients served by medical schemes (*n* = 2111)	Two years	None	Total cost of diabetes (direct and indirect)	Not reported	Provider	US$2330.74 per annum/patient

**Table 2:** Summary of the methods used and cost-effectiveness / economic evaluation studies (*n* = 5)

Author/s and Year	Country	Study design and/or study method	Target population and/or sample size	Duration/ analytical time horizon	Comparator	Intervention type and/or cost items	Intervention effectiveness outcome/s	Perspective	Cost in 2024 US$ and unit of cost	CER, US$/QALYs or unit cost (in 2024 US$)
Bekele et al. (2021) [34]	Ethiopia	Markov model for T2DM disease progression with five health states	A population with controlled type 2 diabetes with one of the second-line treatments (hypothetical age above 40, lifetime)	One year	Only metformin	Saxagliptin plus glibenclamide as a second-line therapy added to metformin	Metformin was more costly and less effective than metformin plus glibenclamide	Provider	US$367/unit cost/annum/patient	US$2727/DALY Averted
Juarez -Garcia et al. (2012) [35]	South Africa	Lifetime simulation model	Patients with type 2 diabetes (*n* = 585)	One year	Sulphonylureas plus Metformin	Saxagliptin plus Metformin	Low incidence of hypoglycaemic events and moderate reduction in complications in the intervention cohort	Provider	Not reported	US$6294.13/QALY gained. Per patient per lifetime
Mash et al. (2015) [36]	South Africa	A randomised controlled trial. Markov micro-simulation model	All uninsured people with type 2 diabetes attending 17 selected community health centers in Cape Town metropolitan area (*n* = 710)	One year	No intervention (*n* = 860)	Group education program	Reduction of blood pressure (systolic by 4.65 mmHg & diastolic by 3.30/mmHg	Provider/ Cost per QALY gained	Not reported	US$2363.40/ICER/ QALY gained
Volmink et al. (2014) [37]	South Africa	Probabilistic modelling utilised for incremental cost-effectiveness ratio analysis	Secondary data used in the model design with total participants (*n* = 8176)	One year	Usual public sector practice clinical model based on the 2002 SEMDSA guidelines	CEA is adapting a private-sector diabetes management programme (DMP) to the South African public sector.	Not reported	Provider	The annual per capita cost of the intervention model was US$932.42, while that of the comparator was US$774.23, a cost increase of 20.3%.	US$1309.25/ICER/ QALY gained
Abide et al. (2013) [38]	Nigeria	A randomised controlled study, activity-based costing	Type 2 diabetes patients who were on hypoglycaemic therapy (*n* = 220)	One year	Usual care	Pharmaceutical care	Not reported	Patients	Not reported	US$742/annum per QALY gained

### Characteristics of included studies

The included studies employed a range of methodological designs, including retrospective case note reviews, cross-sectional cost analyses, randomised controlled trials, quasi-experimental studies, and economic modelling approaches such as Markov models and lifetime simulation models. These studies assessed the cost of illness, evaluated cost-effective interventions, examined disease progression, and analysed treatment adherence and economic outcomes.

The sample sizes range from 33 to 55,000 participants with a median sample size of 605, and in some cases, secondary data with a total of 8,176 participants were used for modelling purposes. Several studies did not report detailed demographic characteristics such as age distribution or sex proportions, while modelling studies focused primarily on simulated lifetime cohorts rather than observed participant characteristics. Where information such as study perspective, participant characteristics, or costing year was unavailable in the original publication, this is indicated as “Not reported” in Tables 1 and 2.

Of the 46 countries in Sub-Saharan Africa, studies on the cost or cost-effectiveness of T2D management were identified in only eight countries, which are Nigeria [7], South Africa [4], Mali [2], Ghana [1], Senegal [1], Ethiopia [1], Kenya [1], and Uganda [1]. As summarised in Tables 1 and 2, five of the included 18 studies conducted cost-effectiveness analyses, reporting outcomes such as cost per QALY gained or DALY averted. Costing perspectives were largely from the provider’s perspective [*n* = 9 studies], followed by patients’ perspectives [*n* = 7], and societal level [*n* = 2] costing research. Most studies evaluated direct and indirect medical costs, outpatient medical costs, and management of referred cases. The cost-effectiveness of saxagliptin plus metformin and saxagliptin plus glibenclamide as a second-line therapy added to metformin and group education programme intervention were assessed in Ethiopia and South Africa, respectively [31, 34]. One study assessed the cost-effectiveness of pharmaceutical care intervention versus usual care (UC) in managing T2D in Nigeria [38] (Table 2).

### Cost outcomes

Cost estimates varied substantially across studies due to differences in costing perspectives, healthcare settings, and cost components included in the analyses. Most studies adopting a provider perspective estimated the direct annual cost per patient of T2D management, covering routine consultations, medications, diagnostic and monitoring tests, and follow-up care [17, 30, 32–34]. Two studies also included indirect costs such as productivity losses, transportation, and caregiver time [18, 30].

In Nigeria, a facility-based study reported an annual direct healthcare cost of US$799.24 per patient, of which 80% was attributed to medications and diagnostic tests [17]. Similarly, a cross-sectional study in Ghana estimated an average annual provider cost of US$498.40 per patient, with medications accounting for 71% of the total [31]. In South Africa, costs for patients with controlled T2D receiving essential chronic treatment were US$986.60 per patient annually, reflecting the inclusion of routine laboratory testing, chronic medication, and clinical follow-up [32]. Another South African study focusing on patients enrolled in private medical schemes reported a substantially higher annual cost of US$2,330.74 per patient, driven by more expensive medications and specialist consultations [33].

Across other countries, direct annual costs of controlled T2D management ranged from US$367 per patient in Ethiopia [34] to US$799.24 per patient in Nigeria [17]. A Nigerian retrospective study including both direct and indirect costs reported an average total annual cost of US$508.37 per patient [18]. Meanwhile, a Kenyan study on uncontrolled T2D patients reported US$46.86 per outpatient visit per patient, covering consultation, diagnostic tests, and pharmacotherapy regimens [28].

From the patient perspective, financial burdens varied widely. In Nigeria, patients faced an out-of-pocket expenditure of US$502.63 per year [25], while in Mali, annual costs ranged from US$249.20 for uncomplicated diabetes to US$337.50 for complicated cases [26]. In a pharmacist-led educational intervention, mean direct costs per patient rose slightly from US$221 to US$225 six months post-baseline (equivalent to US$446 per year) due to increased medication use and follow-up. Nevertheless, the intervention improved adherence, HbA1c, and systolic blood pressure outcomes [24]. Costs varied across studies by perspective (provider vs. patient), inclusion of indirect costs, disease control status, and setting-specific input costs.

Several studies reported costs associated with specific management approaches (e.g., education, digital adherence support, and medication choice) in addition to routine care costing [17, 23, 29, 32]. Across Senegal, Nigeria, and Uganda, these analyses provided insight into the financial implications and effectiveness of alternative care models. In Senegal, the mDiabetes programme delivering tailored lifestyle and medication-adherence messages via SMS proved cost-effective, reducing HbA1c levels over three months at an implementation cost of US$3.80 per patient [23].

In Nigeria, a patient-focused analysis highlighted the economic advantage of generic antidiabetic medications, saving approximately US$0.32 per daily dose compared with branded options [17]. Another study at a tertiary teaching hospital estimated a monthly direct cost of US$55.35 per patient [23]. Conversely, a Ugandan retrospective study reported US$12.60 per visit for outpatient clinic care, reflecting high unit costs in resource-limited settings [29].

Two studies from Mali adopted a societal perspective to capture the broader economic burden of T2D [26, 27]. One estimated median direct medical cost of US$249.20 per patient for uncomplicated diabetes and US$337.50 for complicated cases [27]. The other reported an average annual total cost of US$1,384 per patient, encompassing direct medical costs (inpatient stays, consultations, laboratory tests, medications), direct non-medical costs (transportation and caregiver payments), and indirect costs (productivity losses due to illness and caregiving) [27]. Accounting for this additional cost categories substantially increased total T2D management costs, illustrating the heavy financial burden on households and society at large.

### Cost-effectiveness outcomes

Five studies assessed the cost-effectiveness of T2D interventions, reporting outcomes in terms of cost per QALY gained or DALY averted [34–38]. An activity-based costing study in Nigeria applied a cost-effectiveness acceptability curve and found that a pharmaceutical care intervention was more cost-effective than usual T2D care, with an incremental cost-effectiveness ratio (ICER) of US$742 per QALY gained, below the national cost-effectiveness threshold [38].

In South Africa, a structured group education programme delivered by mid-level healthcare workers at community health centres was cost-effective from the provider perspective, yielding an ICER of US$2,363.40 per QALY gained [36]. Another South African study using secondary data evaluated the adoption of a private-sector capitation-based T2D management model in the public sector and reported an ICER of US$1,309.25 per QALY gained, well below the country’s WTP threshold [37].

A lifetime simulation study of pharmacologic therapy projected that adding saxagliptin to metformin as a second-line treatment improved QALYs at an incremental cost of US$6,294 per QALY gained over a patient’s lifetime [35]. However, in Ethiopia, saxagliptin was not considered cost-effective, with an estimated cost-effectiveness ratio of US$2,727 per DALY averted, exceeding 50% of the national GDP per capita (US$953), the accepted WTP threshold [34]. The study also reported that metformin monotherapy resulted in higher costs and poorer health outcomes compared with combination therapy using metformin plus glibenclamide [34].

## Discussion

This review highlights substantial variability in the cost and cost-effectiveness of T2D management, largely driven by differences in costing perspective, service package composition, and health-system financing arrangements. However, it is important to note that the available evidence is drawn from only eight countries, and therefore findings should not be interpreted as representative of all Sub-Saharan African health systems.

A key implication of these findings is that global cost-effectiveness evidence for T2D interventions cannot be assumed to transfer directly to SSA settings. Even when clinical effectiveness is similar, value-for-money depends on local unit costs, medicine procurement costs, service delivery platforms, and the degree of financial risk protection. In many SSA contexts, higher out-of-pocket payments, fragmented access to diagnostics, and intermittent medicine availability can materially change both the costs incurred and the health gains realised at scale. As a result, interventions that appear cost-effective in high-income settings may not remain affordable or scalable in SSA without adaptation to local delivery and financing constraints.

This distinction matters because economic evaluations are not purely about biological response; they reflect how care is delivered and financed. For example, education and digital adherence support interventions align with broader global evidence, but their effectiveness in SSA depends on patient literacy, continuity of care, availability of medicines, and workforce capacity. Similarly, pharmacologic intensification may show clinical benefit but can be financially unrealistic where second-line therapies are not consistently available or where household payments dominate total spending.

Provider-perspective estimates consistently identified medicines (up to 74%) and diagnostics as the dominant cost drivers, reflecting the ongoing affordability and procurement challenges in many SSA health systems [32, 34]. These costs generally reflect routine consultations, diagnostic tests, and pharmacologic therapy consistent with the WHO Package of Essential Noncommunicable Disease Interventions (WHO PEN) and IDF guidelines. High medication costs relative to other inputs may reflect procurement challenges, limited availability of generic alternatives, or differences in medicine pricing policies across settings.

Two low-cost behavioural interventions, the pharmacist-led education programme and Senegal’s mDiabetes SMS-based initiative demonstrated measurable improvements in glycaemic control and treatment adherence at minimal additional cost [23, 24, 32]. These results align with global evidence that patient-centred education and digital adherence support are cost-effective strategies within resource-constrained health systems.

At the societal level, findings from Mali revealed total annual costs of T2D management that exceeded per-capita GDP, reflecting a substantial economic burden [27]. Including indirect costs such as productivity losses and caregiver time substantially increased total costs, demonstrating that the broader economic consequences of T2D extend beyond the health sector.

Cost-effectiveness analyses further support prioritising interventions that optimise both clinical and economic outcomes. Pharmaceutical care interventions in Nigeria and structured group education in South Africa were consistently cost-effective relative to local willingness-to-pay thresholds [36–38]. Pharmacologic intensification strategies showed mixed economic results. In Ethiopia, the addition of saxagliptin to metformin exceeded the national willingness-to-pay threshold when evaluated using DALYs [34], whereas other modelling studies using QALYs reported different cost-effectiveness conclusions [35].

This cost and cost-effectiveness of managing T2D in the SSA study synthesised significant challenges that warrant careful consideration. Most included studies focused on direct and indirect costs associated with T2D management. The cost of medications dominated as a significant total direct cost item of T2D management in SSA [17, 29, 30]. This supports the previous cost of illness analysis of the T2D study of the LMICs, which reported that the expense of medications drove the direct [6, 39]. There has been a call for urgent intervention to reduce the cost of medications used for T2D management and its comorbidities; with little attention given, this burden continues to affect populations in SSA [38]. For example, 94.3% of the study participants could not afford medications in Uganda [29], which highlights a significant healthcare accessibility and affordability issue, potentially leading to adverse health outcomes due to the inability to access essential medications. Similarly, in Nigeria, the patient-borne costs for follow-up care in tertiary-level hospitals were more than three times higher than the country’s minimum wage of US$125 per month [23], which can deter individuals from seeking timely medical attention, potentially resulting in delayed diagnoses and inadequate treatment. With the rising prevalence of T2D, such direct costs will only grow if current care regimes are unchanged and tracing undiagnosed cases is improved in SSA [40].

The studies in this review highlight the significant societal financial distress caused by managing T2D in SSA. Notably, In a study from Nigeria, the estimated annual cost of T2D management approached 88% of national per capita income, illustrating the substantial financial burden faced by households [17]. Furthermore, the case study of Mali serves as a stark reminder of the complex financial dimensions surrounding T2D management, with annual societal costs surpassing twice the country’s GDP per capita [27]. Available evidence also suggests that individuals with diabetes incurred costs nearly four times higher than those without [27], exceeding comparisons in developed nations. Comprehensive approaches are crucial, including government-led initiatives such as free or subsidised T2D management programmes and proactive preventive advocacy campaigns [36]. Additionally, promoting awareness about the benefits of health insurance schemes is essential in mitigating the financial burden, particularly in low-income countries. Ultimately, the affordability of diabetes treatment remains a pressing concern, necessitating innovative strategies to ensure equitable access to care for vulnerable populations in SSA.

The high rate of out-of-pocket healthcare expenditure in SSA has been widely documented [8], and the management of T2D complications further amplifies this financial strain. Evidence from this review highlights the need for health system–driven programmes and interventions to reduce this burden. For instance, a Nigerian study found that catastrophic health expenditure affected all socio-economic groups, with the poorest quartile experiencing the highest incidence [25]. This financial vulnerability is exacerbated by delayed diagnosis and the cumulative costs of long-term treatment, which together intensify the fiscal pressure on both households and the health sector [39]. Addressing these challenges requires a comprehensive healthcare approach that strengthens prevention, early detection, and effective clinical management within existing service platforms. Integrating affordable screening, improving access to essential medicines, and expanding financial protection mechanisms can reduce the cost burden on patients while improving health outcomes across SSA.

Where assessed, T2D management programmes and interventions in SSA were found to be cost-effective in selective settings [35, 37, 38]. The review also highlights the need to distinguish pharmacologic effectiveness from economic value. While first-line therapies such as metformin are widely supported in global guidelines and are generally considered clinically effective, the relative value-for-money of intensification strategies is highly context dependent. Differences in costs, treatment pathways, baseline risk profiles, and service access can shift incremental costs and outcomes. Therefore, even where clinical benefits are transferable across populations, including individuals of African ancestry, economic conclusions and affordability constraints must be assessed within each SSA setting. For example, structured group education programmes and pharmaceutical care interventions demonstrated favourable cost-effectiveness profiles relative to usual care in Nigeria and South Africa [36, 38]. In contrast, pharmacologic intensification strategies showed mixed economic results. While the addition of saxagliptin to metformin improved health outcomes in modelling studies, its incremental cost per QALY exceeded context-specific thresholds in Ethiopia and was sensitive to national income levels and willingness-to-pay assumptions [34, 35]. These findings reinforce the importance of aligning second-line treatment choices not only with clinical efficacy but also with affordability and country-specific economic benchmarks.

Given that more than half of SSA countries are classified as low-income, the application of appropriate willingness-to-pay thresholds is essential [15, 41]. For instance, one Ethiopian study adopted 50% of GDP per capita as a reference threshold, reflecting growing recognition that conventional WHO-CHOICE benchmarks may overestimate affordability in low-resource settings [35]. Incorporating patient perspectives into cost-effectiveness analyses and explicitly accounting for affordability constraints would further strengthen decision-making [39, 41]. This approach aligns with the WHO call to integrate diabetes management with broader social determinants of health [14].

## Methodological issues and limitations

Some methodological variation across studies was expected given differences in study objectives and analytical approaches. However, incomplete reporting of study perspective, cost components, and data sources limited comparability across studies.

Approximately 60% of the included studies adopted a provider or health system perspective, while relatively few examined patient or societal costs. Evidence on the total societal cost of managing T2D in SSA therefore remains limited, suggesting that the full economic burden of the disease may be underestimated. Only one country provided cost estimates from a societal perspective that incorporated indirect costs such as productivity losses and caregiver time. Excluding these broader costs may underestimate the true economic burden of T2D on households and society.

Another limitation relates to the geographical concentration of evidence. Of the included cost studies, eight were conducted in Nigeria, highlighting the limited availability of cost and cost-effectiveness evidence from other SSA countries. This concentration restricts the generalisability of findings and underscores the need for more diverse and context-specific economic evaluations across the region.

Substantial heterogeneity was also observed in costing methodologies. Studies differed in their costing approaches (e.g., bottom-up versus top-down estimation), cost components included, and the costing years used. Although this review standardised reported costs to 2024 US dollars, differences in original price years, inflation adjustments, and exchange rate assumptions may still influence comparability across studies.

The cost-effectiveness studies included in this review also demonstrated methodological variability. Different outcome measures were used, including cost per QALY gained and cost per DALY averted, which complicates direct comparison across analyses. In addition, studies employed different modelling structures, data sources, and willingness-to-pay thresholds. Some analyses relied on primary clinical data, while others used secondary datasets or simulation models, introducing variation in data quality and assumptions.

Taken together, these methodological differences limited the ability to conduct quantitative pooling of results and required the use of narrative synthesis. Future research should aim to generate more standardised and contextually relevant economic evidence on T2D management in SSA, including broader societal perspectives and consistent methodological reporting.

## Conclusions and policy implications

This review highlights the substantial economic burden of T2D management in SSA, particularly for households with limited financial protection. Despite the growing prevalence of diabetes in the region, only five studies evaluated the cost-effectiveness of T2D interventions, indicating a significant evidence gap for economic decision-making in chronic disease management.

Another important finding is the limited consideration of societal costs in existing studies. Most analyses adopted a provider or health-system perspective, while only one out of the two studies incorporated broader societal costs such as productivity losses and caregiver time. Consequently, the true economic burden of T2D on households and national economies is likely underestimated. Future studies should incorporate societal perspectives to provide a more comprehensive assessment of costs.

The available evidence suggests that behavioural and service-delivery interventions, including structured education programmes, pharmaceutical care models, and digital adherence support tools, may represent promising strategies for improving diabetes outcomes in resource-constrained settings. However, the evidence remains limited and context-specific, highlighting the need for further economic evaluation.

Future research should prioritise economic evaluations embedded within routine diabetes programmes, including cost-of-illness studies, cost-effectiveness analyses, and budget impact assessments. Generating locally relevant economic evidence will be essential for guiding resource allocation, strengthening financial protection, and supporting the development of scalable diabetes management strategies across diverse SSA health systems. 

## Data Availability

All data analysed during this study were obtained from previously published studies and are included in this article and its supplementary materials.

## Appendix

**Table 3:** Detailed search strategies for the listed domains

Medline via PubMed	#1 Search: (Diabetes Mellitus, Type 2[Mesh Terms]) OR (Type 2 Diabetes OR Diabetes Mellitus Type II OR Diabetes OR DMT2 OR Diabetes Mellitus Type 2) #2 (Weight Loss OR Weight Reduction Programs[MeSH Terms]) OR Dietary Education OR Dietary Intake OR Risk Factors OR Risk Reduction OR Risk Reduction Behaviour OR Community Networks OR Community Organization And Administration OR Health Promotion OR Alternative Subjects OR Life Style OR Lifestyle OR Intervention OR Interventions OR Patient Education OR Administration OR Population Surveillance OR Epidemiology OR Behaviour Education OR Behavior Education OR Behavioural Intervention OR Glucose Tolerance OR Fasting Glucose OR Glucose Homeostasis OR Behavioural Intervention OR Nutrition Education OR Lifestyle Intervention OR Nutritionist OR Registered Dietitian OR Registered Dietician OR Physical Activity OR Diabetes Prevention OR Diabetes Control OR Diabetes Management OR Therapy OR Preventive Intervention OR Weight Check OR Stress Management OR Alcohol Cessation OR Social Support OR Nicotine Reduction OR Cigarette Smoking OR Treatment OR Treatments OR Management OR Managements OR Medication OR Medications OR Drug OR Drugs OR metformin OR Life-therapy OR Surveillance OR Food Quality OR Patient Care Management OR Patient Care Planning OR Patient Care Team OR Comprehensive Health Care OR Self Care OR Patient Participation OR patient education OR Reminder Systems OR Information Systems OR Decision Support Systems OR Clinical OR Information Systems OR Decision Support Systems OR Decision Making OR Computer-Assisted OR Ambulatory Care Information Systems OR SMS Text OR Digital Messaging OR Text messaging OR Reminder OR comprehensive lifestyle intervention OR Adiposity OR Weight reduction Program OR Weight Loss OR Weight management OR Reduce Obesity OR Fat Reduction OR Psychological Stress OR Sleep Duration Medical Nutrition therapy OR Pharmacologic Therapy OR hyperglycemia OR Glucagon-like Peptide 1 Receptor OR GLP-1 OR Dual Glucagon-like Peptide 1 Receptor OR Dual GLP-1 OR Glucose-dependent Insulinotropic Polypeptide OR GIP OR Intensive Lifestyle Modification OR Exercise OR Aerobic Exercise OR Resistance Training OR Psychological Interventions OR Insulin Therapy OR Alcohol withdrawal #3 Search: (Costs And Cost Analysis[Mesh Terms]) OR (Cost OR Cost Effectiveness OR Cost-Effectiveness) OR Health Care Cost OR Health Facility Costs OR Health Expenditures OR Economic Aspects OR Economic Evaluation OR Numerical Data #4 Search: Africa South Of The Sahara [Mesh] OR Sub-Saharan Africa, Angola OR Benin OR Botswana OR “Burkina Faso” OR Burundi OR “Cabo Verde” OR Cameroon OR Cameroun OR “Canary Islands” OR “Cape Verde” OR “Central Africa” OR “Central African Republic” OR Chad OR Comoros OR Congo OR “Cote d’Ivoire” OR “Democratic Republic Of Congo” OR Djibouti OR “Eastern Africa” OR Eritrea OR Eswatini OR Ethiopia OR Gabon OR Gambia OR Ghana OR Guinea OR Guinea-Bissau OR “Ivory Coast” OR Jamahiriya OR Kenya OR Lesotho OR Liberia OR Madagascar OR Malawi OR Mali OR Mauritania OR Mauritius OR Mayotte OR Mozambique OR Namibia OR Niger OR Nigeria OR Principe OR Reunion OR Rwanda OR “Sao Tome” OR Senegal OR Seychelles OR “Sierra Leone” OR “Saint Helena” OR Somalia OR “St Helena” OR “South Africa” OR “Southern Africa” OR Sudan OR Swaziland OR Tanzania OR Togo OR Uganda OR “Western Africa” OR “Western Sahara” OR Zaire OR Zambia OR Zimbabwe OR “South Africa” OR “Southern Africa” OR Sudan OR Swaziland OR Tanzania OR Togo OR Uganda OR “Western Africa” OR “Western Sahara” OR Zaire OR Zambia OR Zimbabwe #5 Search: ((((Diabetes Mellitus, Type 2[MeSH Terms]) OR (Type 2 Diabetes[Text Word] OR Diabetes Mellitus Type II[Text Word] OR Diabetes[Text Word] OR DMT2[Text Word] OR Diabetes Mellitus Type 2[Text Word])) AND ((Weight Loss OR Weight Reduction Programs[MeSH Terms]) OR (Dietary Education[Text Word] OR Dietary Intake[Text Word] OR Risk Factors[Text Word] OR Risk Reduction[Text Word] OR Risk Reduction Behaviour[Text Word] OR Community Networks[Text Word] OR Community Organization[Text Word] AND Administration[Text Word] OR Health Promotion[Text Word] OR Alternative Subjects[Text Word] OR Life Style[Text Word] OR Lifestyle[Text Word] OR Intervention[Text Word] OR Interventions[Text Word] OR Patient Education[Text Word] OR Population Surveillance[Text Word] OR Epidemiology[Text Word] OR Behaviour Education[Text Word] OR Behavior Education[Text Word] OR Behavioural Intervention[Text Word] OR Glucose Tolerance[Text Word] OR Fasting Glucose[Text Word] OR Glucose Homeostasis[Text Word] OR Nutrition Education[Text Word] OR Lifestyle Intervention[Text Word] OR Nutritionist[Text Word] OR Registered Dietitian[Text Word] OR Registered Dietician[Text Word] OR Physical Activity[Text Word] OR Diabetes Prevention[Text Word] OR Diabetes Control[Text Word] OR Diabetes Management[Text Word] OR Therapy[Text Word] OR Preventive Intervention[Text Word] OR Weight Check[Text Word] OR Stress Management[Text Word] OR Alcohol Cessation[Text Word] OR Social Support[Text Word] OR Nicotine Reduction[Text Word] OR Cigarette Smoking[Text Word] OR Treatment[Text Word] OR Treatments[Text Word] OR Management[Text Word] OR Managements[Text Word] OR Medication[Text Word] OR Medications[Text Word] OR Drug[Text Word] OR Drugs[Text Word] OR metformin[Text Word] OR Life-therapy[Text Word] OR Surveillance[Text Word] OR Food Quality[Text Word] OR Patient Care Management[Text Word] OR Patient Care Planning[Text Word] OR Patient Care Team[Text Word] OR Comprehensive Health Care[Text Word] OR Self Care[Text Word] OR Patient Participation[Text Word] OR patient education[Text Word] OR Reminder Systems[Text Word] OR Information Systems[Text Word] OR Decision Support Systems[Text Word] OR Clinical[Text Word] OR Decision Making[Text Word] OR Computer-Assisted[Text Word] OR Ambulatory Care Information Systems[Text Word] OR SMS Text[Text Word] OR Digital Messaging[Text Word] OR Text messaging[Text Word] OR Reminder[Text Word] OR comprehensive lifestyle intervention[Text Word] OR Adiposity[Text Word] OR Weight reduction Program[Text Word] OR Weight Loss[Text Word] OR Weight management[Text Word] OR Reduce Obesity[Text Word] OR Fat Reduction[Text Word] OR Psychological Stress[Text Word] OR Sleep Duration Medical Nutrition therapy[Text Word] OR Pharmacologic Therapy[Text Word] OR hyperglycemia[Text Word] OR Glucagon-like Peptide 1 Receptor[Text Word] OR GLP-1[Text Word] OR Dual Glucagon-like Peptide 1 Receptor[Text Word] OR Dual GLP-1[Text Word] OR Glucose-dependent Insulinotropic Polypeptide[Text Word] OR GIP[Text Word] OR Intensive Lifestyle Modification[Text Word] OR Exercise[Text Word] OR Aerobic Exercise[Text Word] OR Resistance Training[Text Word] OR Psychological Interventions[Text Word] OR Insulin Therapy[Text Word] OR Alcohol withdrawal[Text Word] OR OR[Text Word] OR OR[Text Word]))) AND ((Costs And Cost Analysis[MeSH Terms]) OR (Cost[Text Word] OR Cost Effectiveness[Text Word] OR Cost-Effectiveness[Text Word]) OR Health Care Cost[Text Word] OR Health Facility Costs[Text Word] OR Health Expenditures[Text Word] OR Economic Aspects[Text Word] OR Economic Evaluation[Text Word] OR Numerical Data[Text Word])) AND ((Africa South Of The Sahara[MeSH Terms]) OR (Sub-Saharan Africa, Angola[Text Word] OR Benin[Text Word] OR Botswana[Text Word] OR “Burkina Faso“[Text Word] OR Burundi[Text Word] OR “Cabo Verde“[Text Word] OR Cameroon[Text Word] OR Cameroun[Text Word] OR “Canary Islands“[Text Word] OR “Cape Verde“[Text Word] OR “Central Africa“[Text Word] OR “Central African Republic“[Text Word] OR Chad[Text Word] OR Comoros[Text Word] OR Congo[Text Word] OR “Cote d’Ivoire“[Text Word] OR “Democratic Republic Of Congo“[Text Word] OR Djibouti[Text Word] OR “Eastern Africa“[Text Word] OR Eritrea[Text Word] OR Eswatini[Text Word] OR Ethiopia[Text Word] OR Gabon[Text Word] OR Gambia[Text Word] OR Ghana[Text Word] OR Guinea[Text Word] OR Guinea-Bissau[Text Word] OR “Ivory Coast“[Text Word] OR Jamahiriya[Text Word] OR Kenya[Text Word] OR Lesotho[Text Word] OR Liberia[Text Word] OR Madagascar[Text Word] OR Malawi[Text Word] OR Mali[Text Word] OR Mauritania[Text Word] OR Mauritius[Text Word] OR Mayotte[Text Word] OR Mozambique[Text Word] OR Namibia[Text Word] OR Niger[Text Word] OR Nigeria[Text Word] OR Principe[Text Word] OR Reunion[Text Word] OR Rwanda[Text Word] OR “Sao Tome“[Text Word] OR Senegal[Text Word] OR Seychelles[Text Word] OR “Sierra Leone“[Text Word] OR “Saint Helena“[Text Word] OR Somalia[Text Word] OR “St Helena“[Text Word] OR “South Africa“[Text Word] OR “Southern Africa“[Text Word] OR Sudan[Text Word] OR Swaziland[Text Word] OR Tanzania[Text Word] OR Togo[Text Word] OR Uganda[Text Word] OR " Western Africa“[Text Word] OR “Western Sahara“[Text Word] OR Zaire[Text Word] OR Zambia[Text Word] OR Zimbabwe[Text Word]))OR " Western Africa” OR “Western Sahara” OR Zaire OR Zambia OR Zimbabwe)
*Africa-Wide Information/* *Web of Science/* *Cochrane Library*	S1- Diabetes Mellitus OR Type 2 OR Type 2 Diabetes OR Diabetes Mellitus Type II OR Diabetes OR DMT2 OR Diabetes Mellitus Type 2S2- Dietary Education OR Dietary Intake OR Risk Factors OR Risk Reduction OR Risk Reduction Behaviour OR Community Networks OR Community Organization And Administration OR Health Promotion OR Alternative Subjects OR Life Style OR Lifestyle OR Intervention OR Interventions OR Patient Education OR Administration OR Population Surveillance OR Epidemiology OR Behaviour Education OR Behavior Education OR Behavioural Intervention OR Glucose Tolerance OR Fasting Glucose OR Glucose Homeostasis OR Behavioural Intervention OR Nutrition Education OR Lifestyle Intervention OR Nutritionist OR Registered Dietitian OR Registered Dietician OR Physical Activity OR Diabetes Prevention OR Diabetes Control OR Diabetes Management OR Therapy OR Preventive Intervention OR Weight Check OR Stress Management OR Alcohol Cessation OR Social Support OR Nicotine Reduction OR Cigarette Smoking OR Treatment OR Treatments OR Management OR Managements OR Medication OR Medications OR Drug OR Drugs OR metformin OR Life-therapy OR Surveillance OR Food Quality OR Patient Care Management OR Patient Care Planning OR Patient Care Team OR Comprehensive Health Care OR Self Care OR Patient Participation OR patient education OR Reminder Systems OR Information Systems OR Decision Support Systems OR Clinical OR Information Systems OR Decision Support Systems OR Decision Making OR Computer-Assisted OR Ambulatory Care Information Systems OR SMS Text OR Digital Messaging OR Text messaging OR Reminder OR comprehensive lifestyle intervention OR Adiposity OR Weight reduction Program OR Weight Loss OR Weight management OR Reduce Obesity OR Fat Reduction OR Psychological Stress OR Sleep Duration Medical Nutrition therapy OR Pharmacologic Therapy OR hyperglycemia OR Glucagon-like Peptide 1 Receptor OR GLP-1 OR Dual Glucagon-like Peptide 1 Receptor OR Dual GLP-1 OR Glucose-dependent Insulinotropic Polypeptide OR GIP OR Intensive Lifestyle Modification OR Exercise OR Aerobic Exercise OR Resistance Training OR Psychological Interventions OR Insulin Therapy OR Alcohol withdrawalS3- Costs and cost analysis OR Cost OR cost effectiveness OR cost-effectiveness OR Health care cost OR Health Facility Costs OR Health Expenditures OR Economic Aspects OR Economic evaluationS4- Africa South of the Sahara OR Sub-Saharan Africa, Angola OR Benin OR Botswana OR “Burkina Faso” OR Burundi OR “Cabo Verde” OR Cameroon OR Cameroun OR “Canary Islands” OR “Cape Verde” OR “Central Africa” OR “Central African Republic” OR Chad OR Comoros OR Congo OR “Cote d’Ivoire” OR “Democratic Republic of Congo” OR Djibouti OR “Eastern Africa” OR Eritrea OR eSwatini OR Ethiopia OR Gabon OR Gambia OR Ghana OR Guinea OR Guinea-Bissau OR “Ivory Coast” OR Jamahiriya OR Kenya OR Lesotho OR Liberia OR Madagascar OR Malawi OR Mali OR Mauritania OR Mauritius OR Mayotte OR Mozambique OR Namibia OR Niger OR Nigeria OR Principe OR Reunion OR Rwanda OR “Sao Tome” OR Senegal OR Seychelles OR “Sierra Leone” OR “Saint Helena” OR Somalia OR “St Helena” OR “South Africa” OR “Southern Africa” OR Sudan OR Swaziland OR Tanzania OR Togo OR Uganda OR “Western Africa” OR “Western Sahara” OR Zaire OR Zambia OR Zimbabwe"South Africa” OR “Southern Africa” OR Sudan OR Swaziland OR Tanzania OR Togo OR Uganda OR “Western Africa” OR “Western Sahara” OR Zaire OR Zambia OR ZimbabweS5- S1 AND S2 AND S3 AND S4
*CINHAL*	S1- (MH “Diabetes Mellitus, Type 2”) S2- type 2 diabetes OR diabetes mellitus type II OR diabetes OR DMT2 OR diabetes mellitus type 2 S3- S1 OR S2 S4- Dietary Education OR Dietary Intake OR Risk Factors OR Risk Reduction OR Risk Reduction Behaviour OR Community Networks OR Community Organization And Administration OR Health Promotion OR Alternative Subjects OR Life Style OR Lifestyle OR Intervention OR Interventions OR Patient Education OR Administration OR Population Surveillance OR Epidemiology OR Behaviour Education OR Behavior Education OR Behavioural Intervention OR Glucose Tolerance OR Fasting Glucose OR Glucose Homeostasis OR Behavioural Intervention OR Nutrition Education OR Lifestyle Intervention OR Nutritionist OR Registered Dietitian OR Registered Dietician OR Physical Activity OR Diabetes Prevention OR Diabetes Control OR Diabetes Management OR Therapy OR Preventive Intervention OR Weight Check OR Stress Management OR Alcohol Cessation OR Social Support OR Nicotine Reduction OR Cigarette Smoking OR Treatment OR Treatments OR Management OR Managements OR Medication OR Medications OR Drug OR Drugs OR metformin OR Life-therapy OR Surveillance OR Food Quality OR Patient Care Management OR Patient Care Planning OR Patient Care Team OR Comprehensive Health Care OR Self Care OR Patient Participation OR patient education OR Reminder Systems OR Information Systems OR Decision Support Systems OR Clinical OR Information Systems OR Decision Support Systems OR Decision Making OR Computer-Assisted OR Ambulatory Care Information Systems OR SMS Text OR Digital Messaging OR Text messaging OR Reminder OR comprehensive lifestyle intervention OR Adiposity OR Weight reduction Program OR Weight Loss OR Weight management OR Reduce Obesity OR Fat Reduction OR Psychological Stress OR Sleep Duration Medical Nutrition therapy OR Pharmacologic Therapy OR hyperglycemia OR Glucagon-like Peptide 1 Receptor OR GLP-1 OR Dual Glucagon-like Peptide 1 Receptor OR Dual GLP-1 OR Glucose-dependent Insulinotropic Polypeptide OR GIP OR Intensive Lifestyle Modification OR Exercise OR Aerobic Exercise OR Resistance Training OR Psychological Interventions OR Insulin Therapy OR Alcohol withdrawal S5- (MH “Cost Benefit Analysis”) OR (MH “Health Care Costs”) OR (MH “Health Facility Costs”) OR (MH “Costs and Cost Analysis”) OR (MH “Economic Aspects of Illness”) "Costs and Cost Analysis”) OR (MH “Economic Aspects of Illness”) S6- Costs and cost analysis OR Cost OR cost effectiveness OR cost-effectiveness OR Health care cost OR Health Facility Costs OR Health Expenditures OR Economic Aspects OR Economic evaluation S7- S5 OR S6 S8- Africa South of the Sahara OR Sub-Saharan Africa, Angola OR Benin OR Botswana OR “Burkina Faso” OR Burundi OR “Cabo Verde” OR Cameroon OR Cameroun OR “Canary Islands” OR “Cape Verde” OR “Central Africa” OR “Central African Republic” OR Chad OR Comoros OR Congo OR “Cote d’Ivoire” OR “Democratic Republic of Congo” OR Djibouti OR “Eastern Africa” OR Eritrea OR eSwatini OR Ethiopia OR Gabon OR Gambia OR Ghana OR Guinea OR Guinea-Bissau OR “Ivory Coast” OR Jamahiriya OR Kenya OR Lesotho OR Liberia OR Madagascar OR Malawi OR Mali OR Mauritania OR Mauritius OR Mayotte OR Mozambique OR Namibia OR Niger OR Nigeria OR Principe OR Reunion OR Rwanda OR “Sao Tome” OR Senegal OR Seychelles OR “Sierra Leone” OR “Saint Helena” OR Somalia OR “St Helena” OR “South Africa” OR “Southern Africa” OR Sudan OR Swaziland OR Tanzania OR Togo OR Uganda OR “Western Africa” OR “Western Sahara” OR Zaire OR Zambia OR Zimbabwe ” OR “South Africa” OR “Southern Africa” OR Sudan OR Swaziland OR Tanzania OR Togo OR Uganda OR “Western Africa” OR “Western Sahara” OR Zaire OR Zambia OR Zimbabwe S9- S3 AND S4 AND S7 AND S8
*Scopus*	S1- “Diabetes mellitus” OR “type 2” OR “type 2 diabetes” OR “diabetes mellitus type II” OR “diabetes” OR “DMT2” OR “diabetes mellitus type 2” OR “DMT2” OR “diabetes mellitus type 2” S2- “Dietary Education” OR “Dietary Intake” OR “Risk Factors” OR “Risk Reduction” OR “Behavior” OR “Risk Reduction Behaviour” OR “Community Networks” OR “Community Organization and Administration” OR “Health Promotion” OR “Alternative Subjects” OR “Life-Style” OR “Lifestyle” OR “Intervention*” OR “Patient Education” OR “Administration” OR “Population Surveillance” OR “Epidemiology” OR “Behavioural Intervention” OR “Glucose Tolerance” OR “Fasting Glucose” OR “Glucose Homeostasis” OR “Nutrition Education” OR “Nutritionist” OR “Registered Dietitian” OR “Registered Dietician” OR “Physical Activity” OR “Diabetes Prevention” OR “Diabetes Control” OR “Diabetes Management” OR “Therapy” OR “Preventive Intervention” OR “Weight Check” OR “Stress Management” OR “Alcohol Cessation” OR “Social Support” OR “Smoking” OR “Treatment*” OR “Management*” OR “Medication*” OR “Drug*” OR “Metformin” OR “Life-Therapy” OR “Food Quality” OR “Patient Care Management” OR “Patient Care Planning” OR “Patient Care Team” OR “Comprehensive Health Care” OR “Self-Care” OR “Patient Participation” OR “Information System*” OR “Decision Support System*” OR “Clinical” OR “Decision Support Systems” OR “Decision Making” OR “Computer-Assisted” OR “Ambulatory Care Information System*” OR “SMS Text*” OR “Digital Messaging” OR “Text Messaging” OR “Reminder” OR “Comprehensive Lifestyle Intervention” OR “Adiposity” OR “Weight Reduction” OR “Weight Management” OR “Reduce Obesity” OR “Fat Reduction” OR “Psychological Stress” OR “Sleep Duration” OR “Medical Nutrition Therapy” OR “Pharmacologic Therapy” OR “Hyperglycemia” OR “Glucagon-Like Peptide 1 Receptor” OR “GLP-” OR “Dual Glucagon-Like Peptide 1 Receptor” OR “Dual GLP-1” OR “Glucose-Dependent Insulinotropic Polypeptide” OR “Gip” OR “Intensive Lifestyle Modification” OR “Exercise” OR “Aerobic Exercise” OR “Resistance Training” OR “Psychological Interventions” OR “Insulin Therapy” OR “Alcohol Withdrawal” OR “Weight Loss” S3- “Costs and cost analysis” OR “Cost” OR “cost-effectiveness” OR “cost-effectiveness” OR “Health care cost” OR “Health Facility Costs” OR “Health Expenditures” OR “Economic Aspects” OR “Economic evaluation” OR “Health Facility Costs” OR “Health Expenditures” OR “Economic Aspects” OR “Economic evaluation” S4- “Africa South of the Sahara” OR “Sub-Saharan Africa” OR “Angola” OR “Benin” OR “Botswana” OR “Burkina Faso” OR “Burundi” OR “Cabo Verde” OR “Cameroon” OR “Cameroun” OR “Canary Islands” OR “Cape Verde” OR “Central Africa” OR “Central African Republic” OR “Chad” OR “Comoros” OR “Congo” OR “Cote d’Ivoire” OR “Democratic Republic of Congo” OR “Djibouti” OR “Eastern Africa” OR “Eritrea” OR “eSwatini” OR “Ethiopia” OR “Gabon” OR “Gambia” OR “Ghana” OR “Guinea” OR “Guinea-Bissau” OR “Ivory Coast” OR “Jamahiriya” OR “Kenya” OR “Lesotho” OR “Liberia” OR “Madagascar” OR “Malawi” OR “Mali” OR “Mauritania” OR “Mauritius” OR “Mayotte” OR “Mozambique” OR “Namibia” OR “Niger” OR “Nigeria” OR “Principe” OR “Reunion” OR “Rwanda” OR “Sao Tome” OR “Senegal” OR “Seychelles” OR “Sierra Leone” OR “Saint Helena” OR “Somalia” OR “St Helena” OR “South Africa” OR “Southern Africa” OR “Sudan” OR “Swaziland” OR “Tanzania” OR “Togo” OR “Uganda” OR “Western Africa” OR “Western Sahara” OR “Zaire” OR “Zambia” OR “Zimbabwe"” OR “Mali” OR “Mauritania” OR “Mauritius” OR “Mayotte” OR “Mozambique” OR “Namibia” OR “Niger” OR “Nigeria” OR “Principe” OR “Reunion” OR “Rwanda” OR “Sao Tome” OR “Senegal” OR “Seychelles” OR “Sierra Leone” OR “Saint Helena” OR “Somalia” OR “St Helena” OR “South Africa” OR “Southern Africa” OR “Sudan” OR “Swaziland” OR “Tanzania” OR “Togo” OR “Uganda” OR “Western Africa” OR “Western Sahara” OR “Zaire” OR “Zambia” OR “Zimbabwe” S5- S1 AND S2 AND S3 AND S4
*Google Scholar*	S1- Type 2 diabetes mellitus AND lifestyle intervention AND (cost OR cost-effectiveness) AND Sub-Saharan Africa We reviewed the first 100 search outcomes.
